# Neurological and neuropsychiatric manifestations in hospitalised patients with COVID-19

**DOI:** 10.4102/sajpsychiatry.v29i0.2112

**Published:** 2023-11-13

**Authors:** Gondah Lekpeh, Muneeb Salie, Leigh L. van den Heuvel, Soraya Seedat

**Affiliations:** 1 Department of Psychiatry, Faculty of Medicine and Health Sciences, Stellenbosch University, Cape Town, South Africa

**Keywords:** COVID-19, neurological manifestations, neuropsychiatric manifestations, respiratory, hospitalised

## Abstract

**Background:**

Although literature globally indicates varied neurological and/or neuropsychiatric manifestations (NNM) and complications associated with coronavirus disease 2019 (COVID-19), information about NNM in infected hospitalised patients on the African continent remains limited.

**Aim:**

To describe the presentation of NNM and compare patients with and without NNM considering demographic and clinical profiles, treatment, and outcomes.

**Setting:**

Tygerberg Hospital, Cape Town, South Africa.

**Methods:**

Retrospective medical record review of the first 100 consecutively admitted COVID-19 patients (64 females, mean age 47.6 years) between March and June 2020.

**Results:**

Of the 98 patients included in the analysis, 56.1% had at least one NNM. The most common NNM were myalgia (32.7%), headache (21.4%), loss of smell and/or taste (15.3%), and delirium (10.2%). Patients with and without NNM did not differ with respect to demographic characteristics. Patients with NNM had significantly more constitutional symptoms (*p* = 0.017) and were more likely to have neurological and/or neuropsychiatric comorbid conditions (10.9% vs. 0.0%, *p* = 0.033) than those without NNM. Patients without documented NNM were more likely to have abnormalities on chest X-ray (*p* = 0.009) than those with NNM. Coronavirus disease 2019 related treatment and mortality did not differ between the groups.

**Conclusion:**

Neurological and/or neuropsychiatric manifestations were common in hospitalised patients with COVID-19. The results suggest that while COVID-19 patients with NNM may have less of a respiratory phenotype they nonetheless have equivalent mortality rates.

**Contribution:**

This study highlights the common NNM in patients with COVID-19 admitted to Tygerberg Hospital early in the pandemic and adds to the growing evidence of COVID-19 NNM.

## Introduction

Severe acute respiratory syndrome coronavirus 2 (SARS-CoV-2) emerged in Wuhan City, China, in December 2019.^1,2^ The virus spread rapidly in China and other countries, and was soon characterised as a pandemic.^3^ South Africa remains the most affected country on the African continent with 4 070 434 confirmed cases and 102 595 deaths as at 18 March 2023.^4^

At the outset of the coronavirus disease 2019 (COVID-19) pandemic, published studies detailed respiratory manifestations and treatment outcomes of respiratory and pharmacological interventions.^5,6,7^ Evidence has since emerged revealing neurological and/or neuropsychiatric manifestations (NNM) in patients with COVID-19.^8,9^ Neurological and/or neuropsychiatric manifestations are characterised by headache, loss of smell or taste, myalgia, delirium, or any other neurological or neuropsychiatric symptoms such as seizures, focal neurological deficits, and altered level of consciousness. Studies conducted in the Americas, Asia and Europe have reported that the commonest neurological features in COVID-19 patients include headache, myalgia, anosmia and ageusia, impaired consciousness, psychomotor agitation, daytime sleepiness, and dizziness.^10,11,12^ The few studies, conducted in Northern Africa, have reported NNM among hospitalised patients with COVID-19.^13,14,15^

In this study, the NNM in laboratory confirmed COVID-19 patients admitted to a tertiary hospital early in the pandemic (i.e. in the first 4 months) are described and we compare the demographic and clinical characteristics, treatment and outcomes of SARS-CoV-2 infected patients with and without NNM.

## Research methods and design

### Study design

A retrospective medical record review study was conducted. We retrieved data of the first 100 laboratory confirmed cases of COVID-19 admitted at Tygerberg Hospital from March to June 2020. Laboratory confirmation was defined as a positive result for the SARS-CoV-2 Ribonucleic acid (RNA) reverse-transcription polymerase chain reaction (RT-PCR) assay of nasopharyngeal or oropharyngeal swab specimens.

### Study setting

The study was conducted at Tygerberg Hospital, a 1380-bed public sector tertiary hospital in the Eastern Metropole of Cape Town that provides services to approximately 3.5 million people.^16^ The hospital serves mostly vulnerable populations from densely populated low-income communities and rural areas. Tygerberg Hospital was the first designated COVID-19 treatment centre in the Western Cape province.^17^

### Study sample

The first 100 laboratory confirmed COVID-19 patients admitted at Tygerberg Hospital. The inclusion criteria were:

- Confirmed RT-PCR positive SARS-CoV-2 with results available on the National Health Laboratory Service database.
- Must have been admitted to Tygerberg Hospital between 01 March 2020 to 30 June 2020 for COVID-19 and have complete medical records – either hard copy in the Medical Record Department or electronic copy uploaded on the Enterprise Content Management (ECM) of Tygerberg Hospital (complete medical record entails demographic, admission assessment, treatment, and outcome data).

### Data collection

A thorough literature review was done to ascertain the common NNM in patients with COVID-19. A pilot review of medical records of 10 patients with COVID-19 admitted to Tygerberg Hospital was conducted to understand the design of the medical records and to determine the commonly recorded demographic, clinical, laboratory, treatment, and outcome data. A standardised form was designed to extract the data based on previous studies and available variables in the medical records of patients at Tygerberg Hospital. Data were collected regarding headache, loss of smell and/or taste, myalgia, delirium, focal neurology, altered consciousness, seizures and any other NNM reported in the clinical notes. Because of the retrospective nature of the data and the uncertainty of symptom attribution to COVID-19 or other conditions, any NNM reported during the admission was included. Fatigue as a neurological symptom was not included as it is generally considered to be a constitutional symptom. Patients were defined as having NNM if they had any NNM noted in their records at any point during their admission. The presence of symptoms was recorded and not the duration and severity as these data were frequently not possible to extract from the medical records. The data were obtained from the electronic version of patient medical records uploaded on the ECM website of Tygerberg Hospital. Blood biochemistry results were accessed from the National Health Laboratory System of South Africa website, and radiological images and reports were accessed on the Picture Archiving Communication System (PACS) of the Western Cape Department of Health. Data were retrieved and entered into REDCap.^18^

### Measures and outcomes and/or variables

Participant inclusion was determined by confirmed RT-PCR positive result of SARS-CoV-2 and admission to Tygerberg Hospital for COVID-19. Sociodemographic profile consisted of age in years, self-identified sex (male and female), self-identified ethnicity, and marital status (never married, married or co-habiting, divorced or widowed). All documented clinical signs and symptoms and comorbidities in a patient’s record were retrieved. Non-NNM symptoms and/or manifestations were tallied and grouped into respiratory, gastrointestinal, constitutional, and other symptoms. Comorbidities (co-existing conditions and/or diagnoses) were tallied and grouped into metabolic, respiratory, neurological or psychiatric, and other comorbidities. Vital signs were extracted as they indicated the physiological state of a patient. Arterial blood gas results and all laboratory investigation results were retrieved but only those results related to tests that were carried out in more than 60% of the patients were reported on. With regard to vitals and blood biochemistry results, the most deranged (e.g. the highest and/or lowest recordings) results over the course of admission were retrieved. Except for arterial blood gas results, specifically the partial pressures of oxygen and carbon dioxide which were measured in kilopascal (English), all other blood biochemistry results were in metric system units. Radiological imaging and report of chest X-rays were classified based on whether they demonstrated abnormal findings and the number of abnormalities. Treatment variables included the use of antibiotics, antipyretics, steroids, prophylactic anticoagulants as well as oxygen therapy. Outcomes also included mortality and length of stay in hospital.

### Data analysis

Statistical Package for the Social Sciences (SPSS) version 28 ([computer program]. Armonk (NY): IBM Corporation; 2021) was used to analyse the data. Descriptive numerical data with a normal distribution were described using means and standard deviations (s.d.), whereas non-normal data were described using medians and interquartile ranges (IQRs). Sociodemographic, clinical features, observations, special investigations, treatment, and outcome between patients with and without NNM were compared. Chi-square or Fisher’s exact tests were used to compare categorical outcomes between the groups. When comparing the means of continuous data, the *t*-test was used when the data had a normal distribution and the Mann-Whitney *U*-test when the data did not have a normal distribution. Select post-hoc tests were done to examine particular findings further. Significance was set at a *p*-value of < 0.05; because of the small sample size we also commented on results demonstrating a trend towards significance (*p* < 0.1).

### Ethical considerations

Ethical approval was obtained from the Health Research Ethics Committee of the Faculty of Medicine and Health Sciences, Stellenbosch University (Reference No: S21/07/007_COVID-19) and a waiver of patient consent was granted. Tygerberg Hospital’s Research Committee gave approval to access patients’ medical records. Breach of confidentiality risk was mitigated by removing all names and other personal identifiers from the dataset and allocation of a unique code to each participant. The study was conducted in accordance with the Declaration of Helsinki and all other procedures for the ethical conduct of scientific investigation.

## Results

### Sociodemographic characteristics associated with neurological and/or neuropsychiatric manifestations

Most of the patients were females (*n* = 64, 65.3%) with a mean age of 47.6 years (s.d. 15.5; range 2–76). Of the 100 patients included, two patients younger than 16 years (2 years old and 11 years old) were excluded from further analysis. Of the 98 patients included in the analysis, 55 (56.1%) had at least one (range 0–4) NNM, with 24.5% having two or more NNM. The mean number of NNM was 0.9 (s.d. 1.1). The common NNM were myalgia (32.7%), headache (21.4%), loss of smell and/or taste (15.3%), and delirium (10.2%). Furthermore, 8.2% of patients had other NNM (e.g. insomnia, meningism and movement disorder) and 13.3% had NNM that were considered serious or severe, such as focal neurological deficits, seizures and altered level of consciousness. None of the demographic features (age, sex, and ethnicity) differed significantly between the groups with and without NNM (Table 1).

**TABLE 1:** Comparison of socio-demographic characteristics in patients with and without neurological and/or neuropsychiatric manifestations.

Characteristics	All patients (*N* = 98)							NNM														Test value	*df*	*p*
								No (*N* = 43)							Yes (*N* = 55)									
	Mean	s.d.	Range	Min	Max	*N*	%	Mean	s.d.	Range	Min	Max	*N*	%	Mean	s.d.	Range	Min	Max	*N*	%			
**Age (years)**	47.6	15.5	60	16	76	-	-	49.4	13.3	54	21	75	-	-	47.6	15.5	60	16	76	-	-	0.583†	96	0.561
**Sex**	-	-	-	-	-	-	-	-	-	-	-	-	-	-	-	-	-	-	-	-	-	0.214‡	1	0.644
Male	-	-	-	-	-	34	34.7	-	-	-	-	-	16	32.7	-	-	-	-	-	18	37.2	-	-	-
Female	-	-	-	-	-	64	65.3	-	-	-	-	-	27	62.8	-	-	-	-	-	37	67.3	-	-	-
**Ethnicity**	-	-	-	-	-	-	-	-	-	-	-	-	-	-	-	-	-	-	-	-	-	2.835‡	3	0.418
Black African people	-	-	-	-	-	62	63.2	-	-	-	-	-	30	69.8	-	-	-	-	-	32	58.2	-	-	-
Coloured people	-	-	-	-	-	21	21.4	-	-	-	-	-	9	30.6	-	-	-	-	-	12	32.7	-	-	-
White people	-	-	-	-	-	2	2.0	-	-	-	-	-	1	2.3	-	-	-	-	-	1	1.8	-	-	-
Not stated people	-	-	-	-	-	12	12.2	-	-	-	-	-	2	7.0	-	-	-	-	-	10	18.2	-	-	-
**Marital Status**	-	-	-	-	-	-	-	-	-	-	-	-	-	-	-	-	-	-	-	-	-	4.192‡	3	0.241
Never married	-	-	-	-	-	55	56.1	-	-	-	-	-	22	51.2	-	-	-	-	-	33	60.0	-	-	-
Married or cohabiting	-	-	-	-	-	27	27.6	-	-	-	-	-	11	26.5	-	-	-	-	-	16	29.1	-	-	-
Divorced or widowed	-	-	-	-	-	5	5.1	-	-	-	-	-	2	4.7	-	-	-	-	-	3	5.5	-	-	-
Not stated	-	-	-	-	-	11	11.2	-	-	-	-	-	8	18.6	-	-	-	-	-	3	5.5	-	-	-

df, degree of freedom; s.d., standard deviation; max, maximum; min, minimum; NNM, neurological and/or neuropsychiatric manifestations.

† , t-value;

‡ , Pearson Chi-square.

### Non-neurological and/or neuropsychiatric symptoms and comorbidities associated with neurological and/or neuropsychiatric manifestations

Patients had between one and nine non-neurological and/or neuropsychiatric symptoms (Table 2), grouped into respiratory (range 0–4), gastrointestinal (range 0–4), constitutional (range 0–3), and other (range 0–2) symptoms. Those without NNM were more likely to have any respiratory symptom, demonstrating a trend towards significance (*p* = 0.075), whereas patients with NNM had significantly more constitutional symptoms than those without NNM (*p* = 0.017). When comparing individual symptoms between the groups, we found that patients with NNM were significantly more likely to have weakness and/or malaise (34.5% vs. 4.7%, *p* < 0.001) and upper respiratory tract (URT) symptoms (32.7% vs. 11.6%, *p* = 0.014), such as sore throat, than those without NNM.

**TABLE 2:** Comparison of patients with and without neurological/neuropsychiatric manifestations with regards to non-neurological symptoms and comorbidities.

Characteristics	All patients (*N* = 98)				NNM								Test value	Test statistic	*p*
					No (*N* = 43)				Yes (*N* = 55)						
	Median	IQR	*N*	%	Median	IQR	*N*	%	Median	IQR	*N*	%			
**Symptoms**															
Total number of symptoms	4.0	3.0; 5.0	-	-	3.0	3.0; 4.0	-	-	4.0	2.0; 5.0	-	-	1071†	−0.820§	0.412
Number of respiratory symptoms	2.0	1.0; 3.0	-	-	2.0	2.0; 3.0	-	-	2.0	1.0; 3.0	-	-	1074†	−0.807§	0.420
Any respiratory symptom	-	-	90	91.8	-	-	42	97.7	-	-	48	87.3	3.48‡	1¶	0.075††
Number of gastrointestinal symptoms	0.0	0.0; 1.0	-	-	0.0	0.0; 1.0	-	-	0.0	0.0; 1.0	-	-	1116†	−0.596§	0.551
Any gastrointestinal symptom	-	-	29	29.6	-	-	11	26.5	-	-	18	32.7	0.59‡	1¶	0.442
Number of constitutional symptoms	1.0	1.0; 1.0	-	-	1.0	0.0; 1.0	-	-	1.0	1.0; 2.0	-	-	894†	−2.377§	**0.017**
Any constitutional symptom	-	-	77	76.8	-	-	31	72.1	-	-	46	83.6	1.91‡	1¶	0.167
Number of other symptoms	0.0	0.0; 0.0			0.0	0.0; 0.0	-	-	0.0	0.0; 0.0	-	-	1129†	−0.858§	0.391
Any other symptom	-	-	7	7.1	-	-	2	4.7	-	-	5	9.1	0.72‡	1¶	0.462††
**Comorbidities**															
Total number of comorbidities	2.0	1.0; 3.0	-	-	2.0	1.0; 3.0	-	-	2.0	1.0; 3.0	-	-	1094†	−0.652§	0.514
Any comorbidities	-	-	90	91.8	-	-	42	97.7	-	-	48	87.3	3.483‡	1¶	0.075
Number of metabolic comorbidities	1.0	0.0; 0.2	-	-	1.0	1.0; 2.0	-	-	1.0	0.0; 2.0	-	-	1099†	−0.617§	0.537
Any metabolic comorbidity	-	-	70	71.4	-	-	33	76.7	-	-	37	67.3	1.061‡	1¶	0.303
Number of respiratory comorbidities	0.0	0.0; 0.0	-	-	0.0	0.0; 0.0	-	-	0.0	0.0; 0.0	-	-	1055†	−1.460§	0.144
Any respiratory comorbidity	-	-	15	15.3	-	-	9	20.9	-	-	6	10.9	1.869‡	1¶	0.172
Number of other comorbidities	0.0	0.0; 1.0	-	-	0.0	0.0; 1.0	-	-	0.0	0.0; 1.0	-	-	1179†	−0.028§	0.978
Any other comorbidity	-	-	47	48.0	-	-	21	48.8	-	-	26	47.3	0.024‡	1¶	0.878

Note: Constitutional symptoms consist of fever, malaise, and body weakness. Metabolic comorbidities: hypertension, dyslipidaemia, diabetes mellitus and obesity. Respiratory comorbidities: current and past pulmonary tuberculosis (TB), pneumonia, obstructive sleep apnoea and other chronic lung diseases. Other comorbidities:human immunodeficiency virus (HIV), gastro-intestinal conditions, kidney disease and cardiac disease; Bold values denotes significant *p*-values.

IQR, Interquartile range; NNM, neurological and/or neuropsychiatric manifestations.

† , Mann-Whitney U;

‡ , Pearson Chi-square;

§ , score;

¶ , degrees of freedom;

†† , Fisher’s exact *p*-values are reported for cells that have less than five observations.

It was theorised that weakness and/or malaise was likely associated with the NNM myalgia and that URT symptoms were likely associated with the NNM of loss of taste and/or smell. Therefore, post-hoc tests were conducted to investigate the associations between these symptoms, and statistically significant associations between weakness and/or malaise and myalgia (*p* < 0.001), and between URT symptoms and loss of taste and/or smell (*p* < 0.001), were indeed found.

In the sample, 91.8% had at least one comorbidity (range 0–6), with patients without NNM more likely to have a comorbidity than those with NNM, demonstrating a trend towards significance (*p* = 0.075). Comorbidities (Table 2) were grouped into respiratory (range 0–3), metabolic (range 0–4), and other (range 0–4). Patients with NNM were more likely to have a pre-existing neurological and/or psychiatric disorder (10.9% vs. 0.0%, *p* = 0.033), such as depression, Parkinson’s disease, and dementia. Human immunodeficiency virus (HIV) was found in 20.0% of patients with NNM versus 20.9% of patients without NNM (*p* = 0.968). The only specific pre-existing disease that differed significantly between the groups was tuberculosis (TB), with patients without NNM being significantly more likely to have had TB in their lifetime (18.6% vs. 3.6%, *p* = 0.020).

### Vital signs and special investigations in relation to neurological and/or neuropsychiatric manifestations

There were no significant group differences on any vital signs, although patients without NNM were more likely to have tachypnoea (*p* = 0.065) and a higher respiratory rate (*p* = 0.069), demonstrating a trend towards significance (Table 3). Blood gas values and derangements as well as special investigations did not differ significantly between patients with and without NNM (Table 4). Urea and electrolytes (UE) and full blood counts (FBC) were carried out on all patients and the results did not differ between the groups. C-reactive protein (CRP), lactate dehydrogenase (LDH), Alanine transaminase (ALT), and glycated haemoglobin (HbA1C) were carried out on at least 60% of participants and LDH was more likely to be requested on patients without NNM (*p* = 0.023). However, none of the results differed between the groups. Clinicians were more likely to request Vitamin B12 (25.5% vs. 9.3%, *p* = 0.064) and syphilis serology (25.5% vs. 9.3%, *p* = 0.064) on patients with NNM, demonstrating a trend towards significance. The majority of participants had a chest X-ray (96.9%) and patients without NNM were more likely to have abnormalities on chest X-ray (*p* = 0.010). They also had a greater number of abnormalities (range 0–4, *p* = 0.051), demonstrating a trend towards significance (Table 4).

**TABLE 3:** Comparison of vital signs in patients with and without neurological and/or neuropsychiatric manifestations: Observations (*N* = 98).

Characteristics	All patients (*N* = 98)				NNM								Test value	Test statistic	*p*
					No (*N* = 43)				Yes (*N* = 55)						
	Median	IQR	*N*	%	Median	IQR	*N*	%	Median	IQR	*N*	%			
Highest respiratory rate	30.0	25.5; 38.0	-	-	32.0	28.0; 38.0	-	-	24.0	28.0; 36.0	-	-	929†	−1.821§	0.069
Tachypnoea (RR > 20)	-	-	93	95.0			43	100	-	-	50	90.9	4.119‡	1¶	0.065††
Highest temperature	37.5	36.9; 38.6	-	-	37.3	36.8; 38.4			37.7	37.0; 38.7	-	-	1018†	−1.182§	0.237
Fever (> 37.5°C)	-	-	47	48.0	-	-	19	44.2	-	-	28	50.9	0.437‡	1¶	0.509
Highest heart rate	110.0	101.0; 125.3	-	-	110.0	100.0; 122.0	-	-	110.0	103.0; 126.0			1053†	−0.927§	0.354
Tachycardia (HR > 100)	-	-	75	80.0	-	-	30	69.8	-	-	45	81.8	1.951‡	1¶	0.162
Highest systolic blood pressure	151.5	141.0; 167.0	-	-	153.0	141.0; 174.0	-	-	151.0	140.0; 167.0	-	-	1120†	−0.451§	0.652
Highest diastolic blood pressure	88.0	82.0; 98.0	-	-	89.0	81.0; 97.0	-	-	87.0	84.0; 98.0	-	-	1180†	−0.018§	0.986
Hypertension (systolic ≥ 140/diastolic ≥ 90)	-	-	83	85.0	-	-	38	88.4	-	-	45	81.8	0.800‡	1¶	0.371
Lowest systolic blood pressure	105.0	98.0; 113.3	-	-	105.0	98.0; 113.0	-	-	105.0	99.0; 114.0	-	-	1124†	−0.419§	0.675
Lowest diastolic blood pressure	56.0	53.0; 62.0	-	-	56.0	54.0; 61.0	-	-	56.0	52.0; 62.0	-	-	1082†	−0.724§	0.469
Hypotension (systolic < 90/diastolic < 60)	-	-	64	65.3	-	-	29	67.4	-	-	35	63.6	0.154‡	1¶	0.695
Lowest saturation	89.0	81.8; 92.0	-	-	90.0	85.0; 92.0	-	-	88.0	80.0; 93.0	-	-	1092†	−0.649§	0.516
Saturation below 95%	-	-	82	83.7	-	-	37	86.0	-	-	45	81.8	0.316‡	1¶	0.574

Note: The highest oxygen saturations recorded during the admission were within reference range ≥ 95%; therefore, it was not reported in the table.

NNM, neurological and/or neuropsychiatric manifestations; IQR, Interquartile range.

† , Mann-Whitney U;

‡ , Pearson Chi-square;

§ , Z score;

¶ , degrees of freedom;

†† , Fisher’s exact *p*-values are reported for cells that have less than 5 observations. The highest oxygen saturations recorded during the admission were within reference range ≥ 95%; therefore, it was not reported in the table.

**TABLE 4:** Comparison of special investigations in patients with and without neurological and/or neuropsychiatric manifestations.

Characteristics	All patients (*N* = 98)				NNM								Test value	Test statistic	*p*
					No (*N* = 43)				Yes (*N* = 55)						
	Median	IQR	*N*	%	Median	IQR	*N*	%	Median	IQR	*N*	%			
**Blood gases (*N* = 78)**															
PaO_2_	8.5	7.18; 10.58	-	-	8.9	7.5; 10.0	-	-	8.3	6.8; 11.2	-	-	656†	−0.975§	0.330
P/F ratio	200.1	134.5; 317.6	-	-	182.0	133.1; 328.6	-	-	217.9	135.0; 303.6	-	-	745†	−0.080§	0.936
P/F ratio < 300	-	-	53	68.0	-	-	22	62.9	-	-	31	72.1	0.756‡	1¶	0.385††
PaCO_2_	4.8	4.1; 5.3	-	-	4.8	4.0; 5.3	-	-	4.8	4.1; 5.3	-	-	730†	−0.232§	0.817
**PaCO_2_ categories**	-	-	-	-	-	-	-	-	-	-	-	-	0.788‡	2¶	0.674
Low CO_2_ (< 4.7 kPA)	-	-	34	43.6	-	-	17	48.6	-	-	17	39.5	-	-	-
Normal CO_2_ (4.7–6.0 kPA)	-	-	40	51.3	-	-	16	45.7	-	-	24	55.8	-	-	-
High CO_2_ (> 6.0 kPA)	-	-	4	5.1	-	-	2	5.7	-	-	2	4.7	-	-	-
pH	7.5	7.4; 7.5	-	-	7.5	7.4; 7.5	-	-	7.5	7.4; 7.5	-	-	645†	−1.089§	0.276
**pH categories**	-	-	-	-	-	-	-	-	-	-	-	-	1.812‡	2¶	0.404
Acidaemia (< 7.35)	-	-	5	6.4	-	-	3	8.6	-	-	2	4.7	-	-	-
Normal (7.35–7.45)	-	-	31	40.0	-	-	16	45.7	-	-	15	34.9	-	-	-
Alkalemia (> 7.45)	-	-	42	53.9	-	-	16	45.7	-	-	26	60.5	-	-	-
**UE levels Sodium**	139.0	136.0; 142.0	-	-	139.0	136.0; 142.0	-	-	139.0	137.0; 142.0	-	-	1035†	−0.773§	0.440
Sodium categories	-	-	-	-	-	-	-	-	-	-	-	-	0.000‡	1¶	0.996
Normal (136–145 mmol/L)	-	-	67	69.8	-	-	30	69.8	-	-	37	69.8	-	-	-
Deranged (≤ 135/≥ 146)	-	-	29	30.2	-	-	13	30.2	-	-	16	30.2	-	-	-
**Potassium**	4.5	4.0; 4.9	-	-	4.6	4.2; 4.8	-	-	4.4	3.9; 5.1	-	-	998†	−0.527§	0.598
Potassium categories	-	-	-	-	-	-	-	-	-	-	-	-	4.238‡	1¶	0.062
Normal (3.5–5.1 mmol/L)	-	-	75	80.6	-	-	37	96.2	-	-	38	73.1	-	-	-
Deranged (≤ 3.4 /≥ 5.2)	-	-	18	19.4	-	-	5	9.8	-	-	14	26.9	-	-	-
**Creatinine**	76.0	59.8; 121.0	-	-	76.0	60.0; 127.0	-	-	78.0	59.0; 115.0	-	-	1180†	−0.021§	0.983
Elevated creatine (≥ 91 mmol/L)	-	-	33	33.7	-	-	15	34.9	-	-	18	32.7	0.050‡	1¶	0.823
**Urea**	5.5	3.8; 10.1	-	-	6.6	2.9; 12.4	-	-	5.2	3.9;9.4	-	-	1142†	−0.95§	0.925
Elevated urea (≥ 7.2 mmo/L)	-	-	-	-	-	-	20	47.6	-	-	21	38.2	0.869‡	1¶	0.351
**Highest WCC**	7.7	6.3; 11.7	-	-	8.6	6.7; 12.7	-	-	7.7	5.8; 11.1	-	-	1039†	−1.031§	0.303
Normal range (3.90–12.60 × 10^9^/L)	-	-	-	-	-	-	-	-	-	-	-	-	0.597‡	1¶	0.440
No	-	-	28	28.6	-	-	14	32.6	-	-	14	25.5	-	-	-
Yes	-	-	70	71.4	-	-	29	67.4	-	-	41	74.5	-	-	-
**Highest haemoglobin**	13.2	11.9, 14.1	-	-	12.9	11.6; 14.3	-	-	13.2	12.0; 14.1	-	-	1070†	−0.802§	0.423
Normal range (12.0–15.0 g/dL)	-	-	-	-	-	-	-	-	-	-	-	-	2.041‡	1¶	0.153
No	-	-	40	40.8	-	-	21	48.8	-	-	19	34.5	-	-	-
Yes	-	-	58	59.2	-	-	22	51.2	-	-	36	65.5	-	-	-
**Highest platelet**	285.5	224.8; 441.5	-	-	299.0	241.0; 432.0	-	-	266.0	215.0; 464.0	-	-	1133†	−0.354§	0.723
Normal range (186–454 × 10^9^/L)	-	-	-	-	-	-	-	-	-	-	-	-	0.591‡	1¶	0.442
No	-	-	29	29.6	-	-	11	25.6	-	-	18	32.7	-	-	-
Yes	-	-	69	70.4	-	-	32	74.4	-	-	37	67.3	-	-	-
**CRP done**	-	-	90	93.8	-	-	40	95.2	-	-	51	92.7	0.259‡	1¶	0.695
CRP	130.0	45.0; 247.0	-	-	130	45.0; 236.0	-	-	129	52.0; 248.0	-	-	982†	−0.304§	0.761
Elevated CRP (> 10 mg/L)	-	-			-	-	-	-	-	-	-	-	0.004‡	1¶	1.000
No	-	-	7	7.7	-	-	3	7.5	-	-	4	7.8	-	-	-
Yes	-	-	84	92.3	-	-	37	92.5	-	-	47	92.2	-	-	-
**LDH done**	-	-	63	64.3	-	-	33	76.7	-	-	30	54.5	5.180‡	1¶	**0.023**
LDH	447.0	315.0; 530.0	-	-	451.0	278.0; 574.0	-	-	424.0	340.0; 526.3	-	-	494†	−0.014§	0.989
Elevated LDH (> 190 U/L)	-	-	-	-	-	-	-	-	-	-	-	-	0;876‡	1¶	0.614
No	-	-	4	6.3	-	-	3	9.1	-	-	1	3.3	-	-	-
Yes	-	-	59	93.7	-	-	30	90.9	-	-	39	96.7	-	-	-
**ALT done**	-	-	69	70.4	-	-	32	74.4	-	-	37	67.3	0.591‡	1¶	0.442
ALT	25.0	17.0; 46.0	-	-	24.5	15.5; 40.8	-	-	28.0	17.0; 49.0	-	-	500†	−1.114§	0.265
Elevated ALT (> 35 U/L)	-	-	-	-	-	-	-	-	-	-	-	-	2.321‡	1¶	0.128
No	-	-	43	62.3	-	-	23	71.9	-	-	20	54.1	-	-	-
Yes	-	-	26	37.7	-	-	9	28.1	-	-	17	45.9	-	-	-
**Hb_A1C_ done**	-	-	59	60.2	-	-	27	62.8	-	-	32	58.5	0.214‡	1¶	0.644
Hb_A1C_	6.9	6.0; 9.9	-	-	6.8	6.0; 9.9	-	-	7.0	6.2; 10.3	-	-	427†	−0.076§	0.939
Elevated HbA1C (> 6.5%)	-	-	-	-	-	-	-	-	-	-	-	-	0.000‡	1¶	0.993
No	-	-	24	40.7	-	-	11	40.6	-	-	13	940.7	-	-	-
Yes	-	-	35	59.3	-	-	16	59.3	-	-	19	59.4	-	-	-
**Chest X-ray done**	-	-	95	96.9	-	-	41	95.3	-	-	54	98.2	0.653‡	1¶	0.580
Chest X-ray abnormalities	-	-	-	-	-	-			-	-			6.633‡	1¶	**0.009**
No	-	-	8	8.4	-	-	0	0	-	-	8	14.8	-	-	-
Yes	-	-	87	91.6	-	-	41	100	-	-	46	85.2	-	-	-
Number of chest X-ray abnormalities	2.0	1.0; 3.0	-	-	2.0	1.0; 3.0	-	-	2.0	1.0; 2.0	-	-	858	−1.950	0.051

Note: Bold values denotes significant *p*-values.

NNM, neurological and/or neuropsychiatric manifestations; UE, serum urea & electrolyte; WCC, white cell count; CRP, c-reactive protein; LDH, lactate dehydrogenase; ALT, Alanine transaminase; Hb_A1C_, glycated haemoglobin; IQR, Interquartile range.

† , Mann-Whitney U;

‡ , Pearson Chi-square;

§ , Z score;

¶ , degrees of freedom;

†† , Fisher’s exact *p*-values are reported for cells that have less than 5 observations.

### Treatments and outcome in relation to neurological and/or neuropsychiatric manifestations

Patients with and without NNM did not differ significantly in terms of treatments received for COVID-19 (Table 5). Patients with NNM were more likely to have been referred for specialist neurology and/or psychiatry input (10.9% vs. 0.0%, *p* = 0.033), but were not more likely to have received psychotropic drugs (5.7% vs. 2.5%, *p* = 0.632). Patients with and without NNM did not differ in terms of survival or length of stay in hospital (range 1–84 days).

**TABLE 5:** Comparison of treatment and outcome in patients with and without neurological/neuropsychiatric manifestations.

Characteristics	All patients (*N* = 98)				NNM								Test value	Test statistic	*p*
					No (*N* = 43)				Yes (*N* = 55)						
	Median	IQR	*N*	%	Median	IQR	*N*	%	Median	IQR	*N*	%			
**Supplemental oxygen**															
**Nasal prong and/or facemask**															
No	-	-	26	26.5	-	-	12	27.9	-	-	14	25.5	0.074‡	1¶	0.785
Yes	-	-	72	73.5	-	-	31	72.1	-	-	41	74.5			
**Mechanical ventilation**															
No	-	-	84	85.7	-	-	36	83.7	-	-	48	87.3	0.249‡	1¶	0.618
Yes	-	-	14	14.3	-	-	7	16.3	-	-	7	12.7			
**Other treatment**															
**Antibiotics**															
No	-	-	16	16.3	-	-	5	11.6	-	-	11	20.0	1.238‡	1¶	0.266
Yes	-	-	82	83.7	-	-	38	88.4	-	-	44	80.0			
**Antipyretic**															
No	-	-	28	28.6	-	-	14	32.6	-	-	14	25.5	0.597‡	1¶	0.440
Yes	-	-	70	71.4	-	-	29	67.4	-	-	41	74.5			
**Steroids**															
No	-	-	75	76.5	-	-	31	72.1	-	-	44	80.0	0.840‡	1¶	0359
Yes	-	-	23	23.5	-	-	12	27.9	-	-	11	20.0			
**Anticoagulants**															
No	-	-	21	21.4	-	-	6	14.0	-	-	15	27.3	2.543‡	1¶	0.111
Yes	-	-	77	78.6	-	-	37	86.0	-	-	40	72.7			
**Outcome**															
Survived	-	-	85	86.7	-	-	38	88.4	-	-	47	85.5	0.179‡	1¶	0.673
Deceased	-	-	13	13.3	-	-	5	11.6	-	-	8	14.5			
LOS	7.0	4.0; 12.5	-	-	7.0	5.0; 11.0	-	-	8	3.0; 14.0	-	-	1159†	−0.169§	0.866

NNM, neurological and/or neuropsychiatric manifestations; LOS, length of hospital stay; IQR, interquartile range.

† , Mann-Whitney U;

‡ , Pearson Chi-square;

§ , Z score;

¶ , degrees of freedom.

## Discussion

Observation indicated that NNM was commonly reported in hospitalised patients with acute COVID-19. Respiratory, gastrointestinal, and constitutional symptoms co-occurred with NNM, and most patients had at least one pre-existing condition. Patients with NNM had significantly more constitutional symptoms and underlying neurological and psychiatric disorders than those without NNM. Patients without NNM were more likely to have had pre-existing TB and abnormalities on chest X-ray than those with NNM. Supportive treatment was the mainstay of management, and the average length of hospitalisation was 10.0 ± 11.2 days with 13.3% mortality. Patients with NNM received equivalent COVID-19 supportive treatments and had similar mortality to those without NNM.

This study found that 56.1% of patients had at least one NNM and 13.3% had a severe or serious NNM. A similar retrospective study conducted in Iran among hospitalised patients showed a 63.9% prevalence of neurological symptoms in patients with COVID-19.^10^ The higher prevalence of NNM reported by the Iranian study may potentially be because of those investigators having access to more detailed clinical data, such as scales being used within the hospital environment. Furthermore, studies conducted in hospital and community-based settings in Egypt and Tunisia found a prevalence of 50.2% and 72.1%, respectively, of neurological manifestations among confirmed and probable COVID-19 patients.^13,14,15^ The prevalence found in our study is roughly similar to studies conducted in other settings, although methodological differences limit direct comparisons.

There were no differences between patients with and without NNM in relation to age, sex, ethnicity, and marital status in our study. In a previous study, females were more likely to endorse headache than males.^10^ The researchers did not analyse for sex differences in relation to individual NNM in this study because of the limited sample size, which was also predominantly female. Post-hoc testing revealed that headache did not differ according to sex in our sample. A recent systematic review and meta-analysis assessing sex differences in the prevalence of confirmed cases of COVID-19 showed a higher pooled prevalence in males globally.^19^ However, a South Africa demographic report showed that at about the time period of this study, there were more female (57.8%) compared to male (41.3%) confirmed cases of COVID-19 (57.8%).^20^

The most common NNM noted in this study were myalgia (32.7%), headache (21.4%), loss of smell and/or taste (15.3%), and delirium (10.2%). Several studies have reported frequently occurring NNM linked to COVID-19.^11,12,21,22^ A study by Thapa Magar et al.,^21^ in a sample of 3055 COVID-19 patients also documented a preponderance of similar NNM as observed in our setting, with fatigue (32%), myalgia (20%), smell and taste impairment (21%), and headache (19%) most frequently observed.

It was found that patients with NNM had significantly more constitutional symptoms than those without NNM. This was likely because of an association between myalgia and malaise and/or weakness. Other studies have included fatigue under NNM, which we conceptualised as a constitutional symptom.^21^ Researchers also found that patients without NNM were more likely to have respiratory symptoms, although there was only a trend towards significance. It was postulated, and confirmed, that URT symptoms were associated with the NNM loss of smell and/or taste as infection and inflammation in URT tissues would also be more likely to influence taste and smell.

The majority of patients had at least one comorbid medical condition and patients without NNM were more likely to have a comorbid condition than those with NNM, demonstrating a trend towards significance. Metabolic syndrome related conditions and respiratory conditions were the most common comorbid conditions, and these did not differ between patients with and without NNM. In contrast, Khedr et al.,^13^ demonstrated that pre-existing hypertension, ischaemic heart disease, diabetes mellitus, impaired liver function, impaired renal function, lung disease, and neurological disease are risks factors for the development of neurological manifestations in patients with COVID-19.

The present study found that COVID-19 infected patients with pre-existing neurological and psychiatric disorders were more likely to have NNM recorded in their clinical notes. Patients who had lifetime TB were also found to be less likely to have NNM as compared to those with no history of TB. This isolated finding is contrary to the findings of Khedr et al.^13^ who showed that chest disease was one of the risk factors for development of neurological manifestations in patients with COVID-19. The presence and absence of NNM did not differ with regards to HIV. This finding is consistent with a previous study in the same setting which did not find significant differences between patients with and without HIV in relation to severity and outcome of COVID-19.^17^

Vital signs and special investigations in the majority of patients revealed acute COVID-19 illness as evidenced by tachypnoea, tachycardia, elevated blood pressure, low oxygen saturation (with PF < 300), and elevated CRP and LDH. Studies have shown that lower oxygen saturation and diastolic blood pressure, and higher respiratory rate and blood glucose level in patients with COVID-19 are associated with disease severity and mortality.^23,24,25^ In addition, severe COVID-19 has been linked to neuropsychiatric manifestations and disorders during acute disease and post-COVID-19.^26,27,28^

This study did not find differences between patients who had NNM and those who did not with regards to vital signs including oxygen saturation. However, patients without NNM had higher respiratory rates and were more likely to have tachypnoea, demonstrating a trend towards significance. C-reactive protein and LDH were markedly elevated in the sample overall as reported in other literature.^29,30^ However, the level of CRP and LDH did not differ in patients with and without NNM. Clinicians were, however, more likely to request LDH on patients without NNM. Lactate dehydrogenase is a marker of more severe disease, and it is postulated that this may suggest that clinicians were more likely to consider patients without NNM as being more severely ill and to request LDH on them.

Unlike vital signs and blood biochemistry results, chest X-ray abnormalities were more frequently observed in patients without NNM, and they had more abnormalities on their chest X-ray, demonstrating a trend towards significance. A previous study has linked the degree of chest radiological (lung) abnormalities to the severity of COVID-19 and poor prognosis.^31^ Several studies have linked the severity of COVID-19 to neuropsychiatric manifestations and sequelae.^26,27,32^ Though the indirect link of chest radiological abnormalities and severity of COVID-19 to neuropsychiatric sequelae has been established, we did not find any studies directly linking chest X-ray abnormalities to NNM. Finally, treatment, duration of hospitalisation and outcomes did not differ significantly between patients with and without NNM.

Patients without NNM were more likely to have a ‘respiratory’ phenotype than those with NNM. This is supported by patients without NNM being more likely to have respiratory symptoms, more likely to have had TB, more likely to have tachypnoea and a higher respiratory rate, and more likely to have abnormalities on chest X-ray. Despite this, they were not more likely to be ventilated. However, patients with a poor prognosis may not have been candidates for ventilation. Despite having more severe respiratory features, patients without NNM also did not have a higher mortality rate. Patients with NNM may thus have a phenotype that involves less typical COVID-19 respiratory features but have a similar prognosis and outcome.

### Limitations and strengths

The study was conducted at a single medical centre and only included hospitalised patients; thus, the findings may not be representative of the general population. Moreover, some important clinical features may have been omitted from documentation. For instance, early in the pandemic clinicians may have been more focussed on the typical respiratory symptoms related to COVID-19, and may have paid less attention to NNM. Certain laboratory tests were not done in all the patients. Missing data may limit the generalisability of the study findings. Furthermore, it is important to note that NNM that presented at various points during admission cannot necessarily be attributed to COVID-19 infection. Side effects of treatment or comorbid conditions may have caused NNM.

However, even with these limitations, we assume that the study is a representation of hospitalised COVID-19 patients with NNM in the Western Cape province. To the best of our knowledge, this is the first study reporting NNM in hospitalised patients with COVID-19 in South Africa. Although the sample size is small, the study shows that NNM are common in patients with COVID-19; however, there is a need for other studies in South Africa and on the African continent, and in larger samples, to corroborate these findings.

## Conclusion

Neurological and/or neuropsychiatric Manifestations are common in patients with COVID-19. This study highlights that even during acute illness with COVID-19, features of NNM can be identified. Raising awareness, early identification during- and post-hospitalisation, and monitoring and management of NNM are important to potentially avert medium and long-term sequelae, such as long COVID-19.
